# Design and fabrication of 3D-printed *in situ* crystallization plates for probing microcrystals in an external electric field

**DOI:** 10.1107/S1600576724002140

**Published:** 2024-04-15

**Authors:** Krishna Prasad Khakurel, Michal Nemergut, Veronika Džupponová, Kamil Kropielnicki, Martin Savko, Gabriel Žoldák, Jakob Andreasson

**Affiliations:** a ELI Beamlines Facility, The Extreme Light Infrastructure ERIC, Za Radnicí 835, 25241 Dolní Břežany, Czech Republic; b Center for Interdisciplinary Bio­sciences, Technology and Innovation Park, P. J. Šafárik University, Košice, Slovakia; c Soleil Synchrotron, Saint-Aubin, France; SLAC National Accelerator Laboratory, Menlo Park, USA

**Keywords:** *in situ* X-ray crystallography, external electric fields, 3D printing, crystallization plates, macromolecules

## Abstract

X-ray crystallography is an established tool to probe the structure of macromolecules with atomic resolution. Compared with alternative techniques such as single-particle cryo-electron microscopy and micro-electron diffraction, X-ray crystallography is uniquely suited to room-temperature studies and for obtaining a detailed picture of macromolecules subjected to an external electric field (EEF). The impact of an EEF on proteins has been extensively explored through single-crystal X-ray crystallography, which works well with larger high-quality protein crystals. This article introduces a novel design for a 3D-printed *in situ* crystallization plate that serves a dual purpose: fostering crystal growth and allowing the concurrent examination of the effects of an EEF on crystals of varying sizes. The plate’s compatibility with established X-ray crystallography techniques is evaluated.

## Abstract 2

A 3D-printed *in situ* crystallization plate is presented, which can be used to probe protein microcrystals in an external electric field.

## 1. Introduction

X-ray crystallography is an established technique used to render three-dimensional atomic pictures of macromolecules (Smyth & Martin, 2000 ▸). With the emergence of competing techniques such as single-particle cryo-electron microscopy (Doerr, 2016 ▸) and micro-electron diffraction (Nannenga & Gonen, 2019 ▸; Khakurel *et al.*, 2019 ▸) for obtaining atomic-resolution structures of proteins, the focus of X-ray crystallography has shifted towards the dynamics of the macromolecules, room-temperature studies and *in situ* crystallography (Shoemaker & Ando, 2018 ▸). A particular niche for X-ray crystallography is to gain biophysical insights into the atomic dynamics of macromolecules in the presence of an external electric field (EEF) (Hekstra, 2023 ▸). Interest in understanding electrostatic preorganization during enzyme catalysis and the possibility of controlling enzyme function with an EEF has grown significantly in recent times.

Electric fields are an intrinsic feature of enzymes (Fried & Boxer, 2017 ▸). It has been proposed since the late 1970s that the significant gain in reaction speed in enzyme catalysis can be attributed to electrostatic preorganization in the enzymes (Warshel, 1998 ▸). While the discussion has lasted for decades, it is only recently that several serious experiments to understand macromolecular mechanics in the presence of an EEF have been carried out (Hekstra *et al.*, 2016 ▸). For all experiments to date, atomic pictures of the influence of an EEF on macromolecules have been obtained through single-crystal X-ray crystallography. There are two major limitations to this technique, namely the requirement for large high-quality crystals and the challenges associated with conducting room-temperature studies. These limitations hinder the understanding of the influence of EEFs as they exclude from systematic investigations the wide range of proteins that do not crystallize into large high-quality crystals. In particular, membrane proteins, which occur natively in cell membranes where the electric field is supposed to be high, are typically challenging to grow into high-quality crystals of sizes suitable for single-crystal X-ray crystallography. Consequently there are significant challenges in understanding the atomistic details of their response to an EEF. For proteins that do crystallize to the size and quality suitable for single-crystal X-ray crystallography, challenges remain in obtaining atomic-resolution pictures of the influence of an EEF under physiologically relevant conditions, *i.e.* at room temperature. These limitations can, in some cases, be circumvented by the use of serial crystallography (Pearson & Mehrabi, 2020 ▸).

Traditionally, single-crystal X-ray crystallography is the method of choice to obtain the structures of macromolecules. In this method, a single crystal is exposed to X-rays, and as it rotates along the rotation axis diffraction patterns are recorded (Dauter, 2017 ▸). These diffraction patterns are used to obtain the three-dimensional electron-density distribution of the macromolecules. In the past decade, serial crystallography has emerged as an alternative to single-crystal X-ray crystallography (Standfuss & Spence, 2017 ▸). The technical advantage of serial crystallography is that it removes the necessity for large high-quality single crystals and enables the collection of data sets at room temperature. In serial crystallography, a single diffraction pattern or a small wedge from many small crystals in random orientations is recorded, from which structures can later be obtained.

The ability to obtain structures from protein microcrystals has accelerated the solving of structures of challenging proteins, studying the kinetics of enzymes and the photo-excited dynamics of macromolecules. A comparative study of serial crystallography and traditional single-crystal X-ray crystallography has also suggested minimal radiation damage by the former technique, thus indicating the possibility of detecting subtle changes in the protein dynamics using this method. While microcrystal X-ray diffraction has been routine on various synchrotron macromolecular crystallography beamlines, it has not yet been applied to observe the influence of an EEF on microcrystals. We see a major limitation in the previous experiments applying EEFs to protein crystals in the lack of a proper *in situ* device to grow the crystals and probe them while exposed to the EEF. Here, we introduce the design of a 3D-printed *in situ* crystallization plate for this purpose.

Only a few studies have explored the utilization of 3D-printed crystallization plates. Han *et al.* (2019 ▸) introduced a novel 3D-printed microcrystallization chip designed for versatile and high-efficiency droplet evaporative crystallization processes. This microstructured platform is crafted using advanced 3D printing and holds significant promise for applications across crystallization work, bioengineering and particle drug preparation. The key innovation lies in the chip’s limited interfacial area and its ability to control the crystallization processes. By allowing for different drop locations and evaporative conditions during liquid injection, the chip facilitates flexible and distinct crystallization processeses. The study provides crucial insights into nucleation control, growth mechanisms and crystal formation dynamics at stable evaporative rates. Additionally, it investigates the impact of initial concentration and droplet contact conditions at the triple-phase interface on crystal properties and distributions. The research sheds light on various intriguing phenomena, including ‘coffee ring’ formation during evaporative crystallization, dendritic crystal growth and hydrate crystallization mechanisms. Notably, within the microstructure, the capillary flow of liquid drops plays a significant role in driving crystal distribution and morphology. The paper also discusses the need to rectify incorrect liquid drop placements to enhance process repeatability and efficiency. Applications of this innovative chip extend to the manufacturing of particle drugs and flow chemistry, offering possibilities to advance research and applications in these fields. In another study, Huang *et al.* (2020 ▸) demonstrated the use of 3D-printed holders for fixed-target crystallographic data collection; these holders are designed for *in situ* crystallization, particularly in lipid cubic phases, which is crucial for determining membrane protein structures. There has also been a previous report of data collected from crystals grown in 3D-printed *in situ* plates (Liang *et al.*, 2020 ▸).

In our research, we have employed advanced 3D-printed *in situ* crystallization plates that are equipped to provide the capability of applying an electric field. This innovative setup enables us to conduct data collection seamlessly under serial conditions and at room temperature. We have successfully cultivated crystals and collected valuable data from carefully chosen protein and DNA samples.

## 2. Experiment

The primary goal of this article is to present a new design of an *in situ* crystallization plate in which protein crystals can be grown and subsequently subjected to an EEF. Such *in situ* plates are ideally suited to probing microcrystals in an EEF.

### 2.1. Design of the *in situ* plate

A major limitation in the study of the influence of an electric field on a microcrystal is the lack of a proper custom­izable crystallization plate or sample manipulation technique. No previously used methods are suitable for handling microcrystals and performing the experiments in a serial crystallography mode. To address this problem, we have designed an *in situ* crystallization plate capable of applying an electric field.

**Figure 1:**
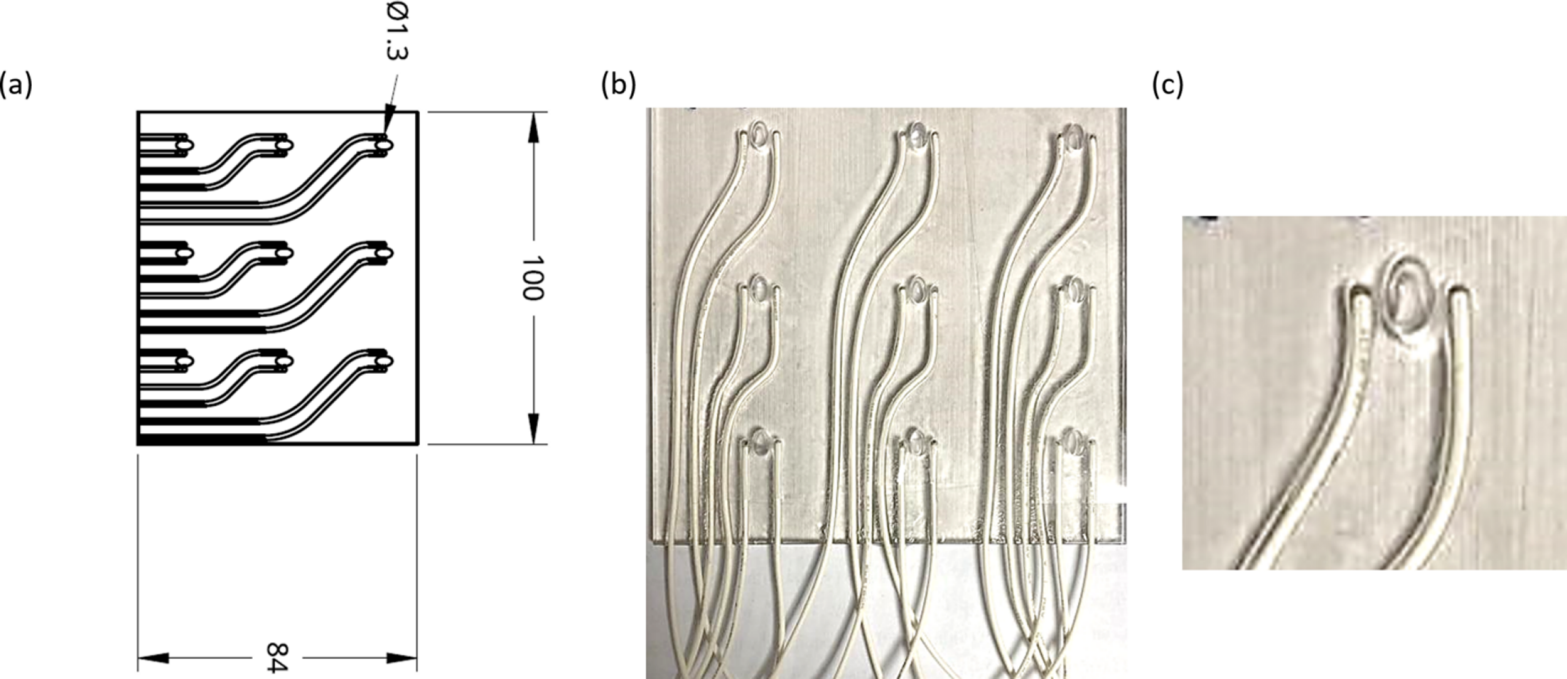
(*a*) A computer-assisted design (CAD) drawing of the *in situ* plate (the measurements in the drawing are in millimetres). (*b*) The physical 3D-printed *in situ* plate, with wires for the application of the EEF. (*c*) An enlarged image of a well in the plate.

The design of the plate is shown in Figs. 1 ▸(*a*) and 1 ▸(*b*). For the initial trials, the number of wells has been limited to nine to ensure that there is no interaction of the electric field between adjacent wells. However, this number is not an upper limit. The dimensions of the plate (8.4 × 10 cm) were adopted to fit the beamline requirements of the Proxima 2A beamline at the SOLEIL synchrotron but can be adapted for different experimental requirements. The grooves in the plates are channels for the wires used to apply the electric field. The width of the grooves has been designed to hold the wires tightly, preventing any movement. In our recent experiments, the wires were also fixed using glue. Each well is an ellipse of *a* = 2 mm and *b* = 3 mm. The choice of the elliptical shape is to facilitate the collection of diffraction from large tilt angles. The depth of each well is 3 mm. An enlarged image of the well in the plate is shown in Fig. 1 ▸(*c*).

### 2.2. Fabrication of the plate

The plate was printed using a Stratasys Objet30 3D printer. The polymer used in printing the plate was VeroClear (polypropylene). For this work, we did not screen printing polymers to minimize the background of the polymer. More research in this direction may further increase the usefulness of the method by determining the polymer with the smallest influence on the crystallization.

Two different types of plates were fabricated to check the background from different substrates. The plates of the first type were printed with integrated wells, with the bottom support of the well being the polymer material. The plates of the second type were printed ‘hole through’ using standard crystallographic *in situ* seals as the bottom support. The wires used in the plate, as shown in Fig. 1 ▸(*b*), are standard wires that can withstand up to 6000 V. With the present design, the EEF in the plate can reach a maximum value of 25 kV cm^−1^. This value is not to be considered as an upper limit but could be increased further by appropriate adaptations of the original design. The use of high-voltage wires and a properly insulated environment can ensure the application of a very high electric field on small crystals. However, the use of wires as electrodes can introduce a non-uniform electric field within the well. A more uniform electric field can be generated using plate electrodes instead of wires. A picture of the plates connected to the high-voltage power supply and of the mounting of the plates on the synchrotron beamline is shown in Fig. S1 in the supporting information.

### 2.3. Crystallization

**Figure 2:**
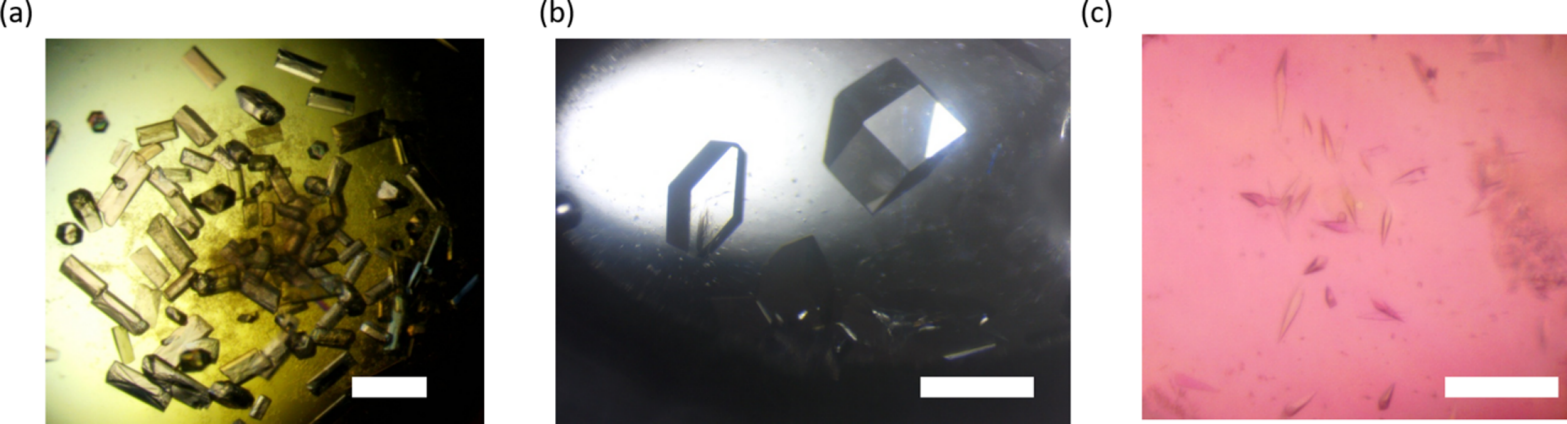
Crystal growth in the 3D-printed *in situ* plate, (*a*) DNA crystals (*b*) lysozyme crystals and (*c*) JTO light chain crystals. The scale bar in the images corresponds to 20 µm.

In order to verify that crystals can be grown in the designed plate, we conducted the crystallization of three different macromolecules: lysozyme, human light chain (JTO) and DNA (Fig. 2 ▸). Proper sealing of the plate was the only condition required to guarantee the growth of these crystals. The crystallization experiments for each of the macromolecules are explained below. The crystallization plates were set up manually. The protein solution and the precipitant were pipetted into each well. With further work on the design, the plates could potentially be developed to support crystallization with robots. This will be pursued in the future. All the crystallization experiments presented in this article were performed using the batch method. The plates were sealed with *in situ* plate seals (MiTeGen, catalogue No. ML-CDSF1-10) made up of 25 µm COC film. There was no noticeable evaporation for a period of a week as the crystallization drops remained intact. Monitoring of evaporation from the plate for a longer period has not been done.

Lysozyme from chicken egg white (Merck Millipore Ltd, Ireland) was gently dissolved in solubilization buffer (20 m*M* sodium acetate pH 4.5) at a concentration of ∼60 mg ml^−1^. Subsequently, the solubilized lysozyme was mixed with the crystallization solution (1.5 *M* NaCl, 100 m*M* sodium acetate pH 4.5) in a 1:1 ratio. Crystallization was carried out in our designed plate by a batch method in which a maximum of 50 µl of premixed protein and precipitant solutions was sealed and left undisturbed. Lysozyme crystals usually appeared within two days.

JTO light chain was crystallized in crystallization buffer (0.2 *M* NH_4_H_2_PO_4_, 20% PEG 3350 pH 8.0) at room temperature according to the published protocol (Morgan *et al.*, 2019 ▸).

To obtain DNA crystals, the Z-DNA hexamer d(CGCGCG) was synthesized and purified by high-performance liquid chromatography (Integrated DNA Technologies). Lyophilized DNA hexamer was dissolved in pure water to a concentration of 50 mg ml^−1^. The crystallization buffer was prepared using dry MES [2-(*N*-morpholino)ethane­sulfonic acid], ammonium sulfate and magnesium acetate dissolved in water to give 2.5 *M* ammonium sulfate, 10 m*M* magnesium acetate and 50 m*M* MES. The final pH of the buffer was 6.1. Crystals were grown by mixing equal volumes of DNA solution and crystallization buffer (Harp *et al.*, 2018 ▸).

## 3. Data collection

### 3.1. Background estimation from the plate

**Figure 3:**
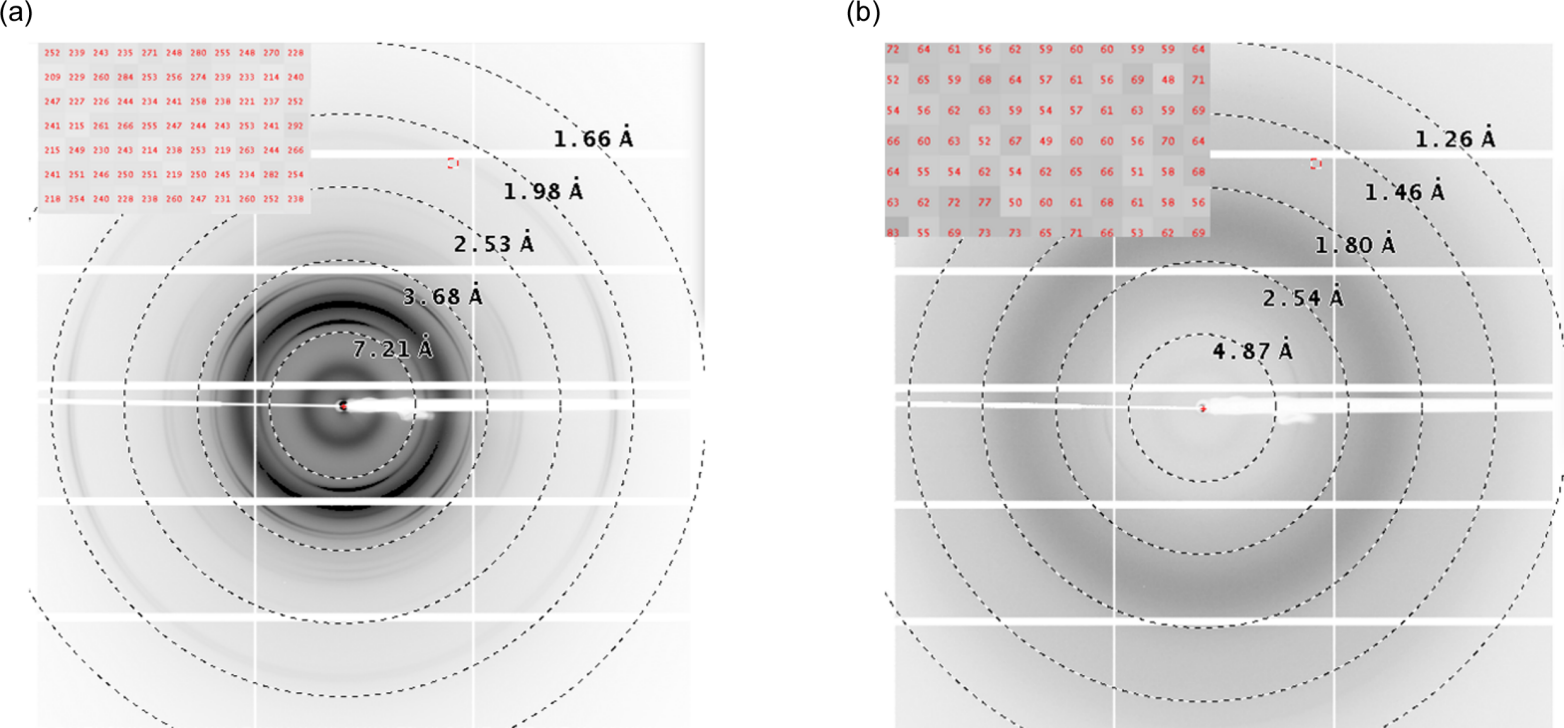
Background signals (*a*) from the MiTeGen *in situ* plate and (*b*) from the 3D-printed *in situ* plate.

Prior to collecting data from the crystals, we performed an experiment to determine the background level from the plate (with polymer material as the bottom support) and compare it with the standard commercially available *in situ* plate (MiTeGen). The background images are shown in Fig. 3 ▸. At low resolution, we did not observe any additional background from the designed plate compared with the commercial *in situ* plate. However, the designed plate has a strong broad peak at ∼3 Å. The background at resolution beyond 3 Å is either lower than or equal to that from the commercial *in situ* plate. The scattering pattern shown in Fig. 3 ▸(*a*) was collected in the presence of the same volume of buffer as was used for crystallization of lysozyme in the plates. Fig. 3 ▸(*b*) shows the background only from the 3D-printed plate, showing the characteristic peak of the polymer used in printing the plate. The inset shows the background photons per pixel under the given conditions. The background from the plates with the standard crystallization *in situ* plate seal as the bottom support showed no significant difference, at either low or high resolution, from the commercial *in situ* plate. A plot showing the azimuthally averaged intensity of the background collected under three different conditions is shown in Fig. S2.

### 3.2. Data set collection

**Figure 4:**
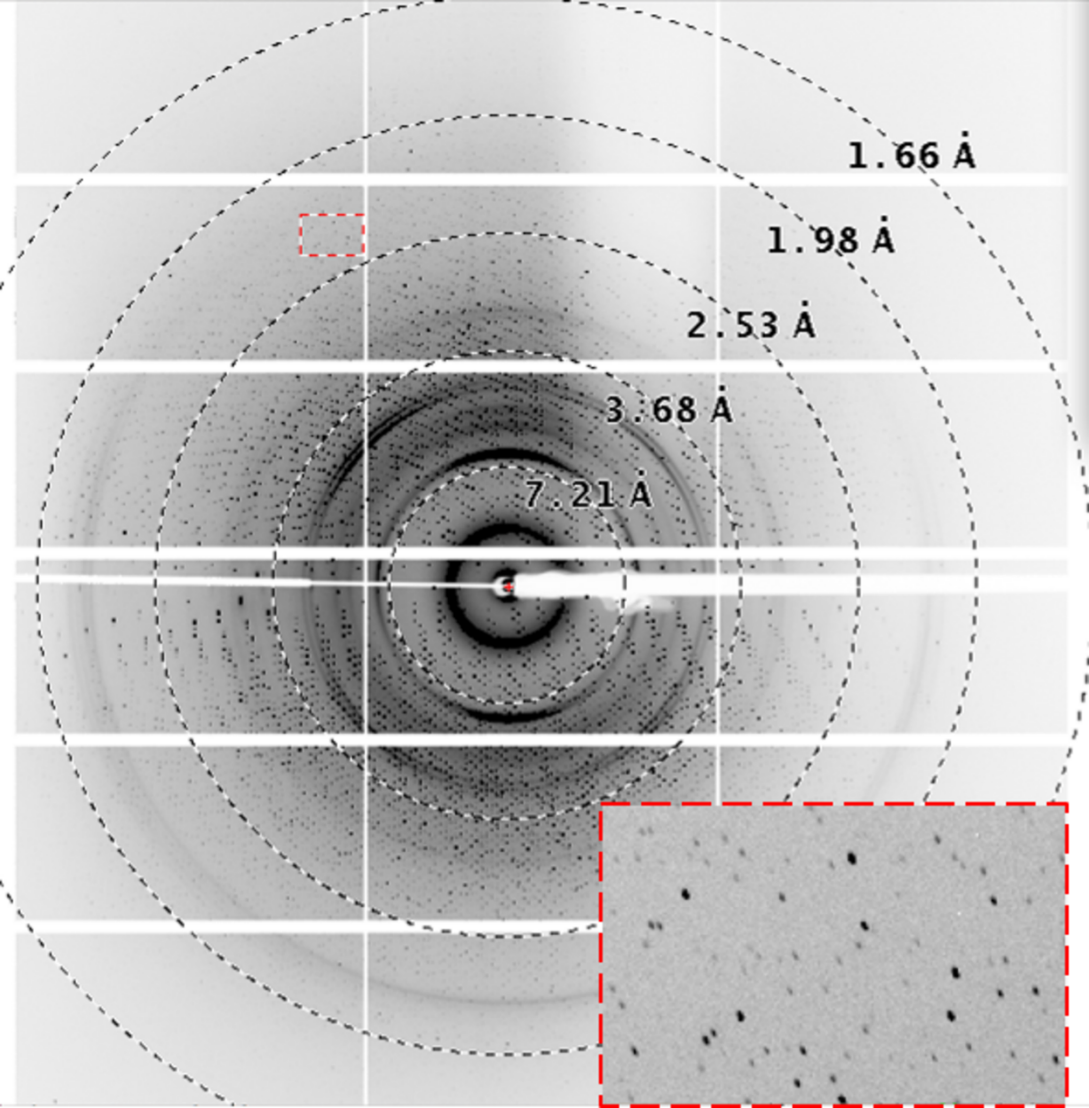
An example of diffraction from lysozyme crystals grown in the 3D-printed *in situ* plate. The inset shows the peaks beyond 2.5 Å in the diffraction pattern and represents an enlargement of the region highlighted by the small red rectangular dashed box in the parent figure.

As a proof-of-principle experiment, we collected data sets from the lysozyme crystal. Data collection was done on the Proxima 2A crystallography beamline at the SOLEIL synchrotron facility. The plates were mounted on the beamline and all the measurements were done at room temperature (295 K). The data were collected using 12.65 keV X-rays with a flux of ∼10^11^ photons s^−1^. The distance between the crystal and the detector was set to 21.6 cm. All the data presented here were collected in an angular range of ±30°. The oscillation range for a diffraction pattern is 0.1°. For each frame, the exposure time was set to 5 ms. The diffraction pattern showed visible peaks up to ∼1.7 Å. An example of a diffraction pattern collected from the crystal grown in the plate is shown in Fig. 4 ▸.

### 3.3. Data reduction

**Figure 5:**
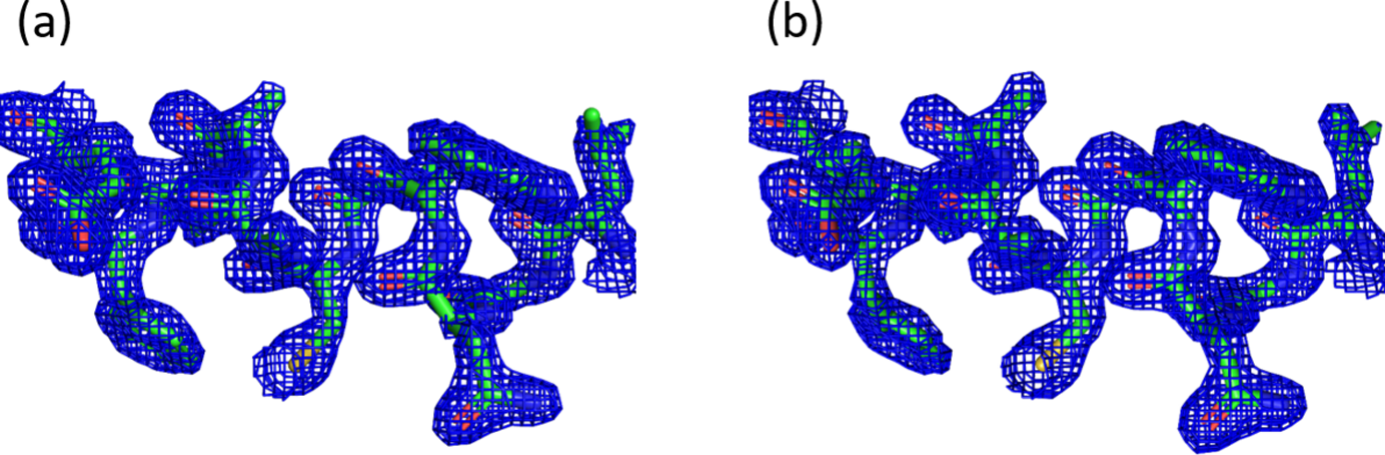
2*mF*_obs_–*DF*_calc_ electron density (contoured at 1σ) for residues 25–35 of (*a*) Lys1 and (*b*) Lys 2.

The data sets collected were reduced using the *XDS* software (Kabsch, 2010 ▸). In this article, we present two data sets named Lys1 and Lys2. Data set Lys1 was collected from two crystals grown on the plates with the polymer support at the bottom of the well, and data set Lys2 was collected from a crystal with the crystallization done with the *in situ* plate seal, both as the bottom support and the top sealing material. The integration and correction were performed using *XDS* and the merging and scaling with *AIMLESS* (Evans & Murshudov, 2013 ▸). The scaled data were phased by *MolRep* (Vagin & Teplyakov, 2010 ▸) using a previously solved high-resolution structure (PDB ID 1dpx; Weiss *et al.*, 2000 ▸) as the input model. The phased model was refined using *REFMAC* (Murshudov *et al.*, 2011 ▸). The statistics for the data reduction and structure solution are summarized in Table 1 ▸. The 2*mF*_obs_–*DF*_calc_ electron-density maps of Lys1 and Lys2 generated using *PyMOL* (Warren L. DeLano, https://legacy.ccp4.ac.uk/newsletters/newsletter40/11_pymol.pdf) are shown in Fig. 5 ▸.

## 4. Conclusion

We have developed and manufactured an *in situ* crystallization plate specifically designed for applying an EEF to microcrystals. The feasibility of this *in situ* plate for crystal growth has been confirmed through the successful crystallization of selected protein and DNA samples, from which the high-resolution structure of the lysozyme sample was determined. Data on DNA and JTO light chain crystals, including the effects of application of an electric field on the crystals, have also been investigated and will be presented in a separate article.

The designed plate can be used for the study of protein crystallization in the presence of an external electric field. We anticipate that the ongoing development and demonstration of the application of this plate for a wide range of proteins will pique the interest of researchers interested in understanding the impact of electric fields on challenging-to-crystallize proteins in a serial crystallography context.

## Supplementary Material

Additional figures. DOI: 10.1107/S1600576724002140/te5134sup1.pdf

## Figures and Tables

**Table 1:** Crystallographic data collection and refinement statistics Values in parentheses refer to the highest-resolution shells.

	Lys1	Lys2
Data collection		
Wavelength (Å)	0.98	0.98
Space group	*P*4_3_2_1_2	*P*4_3_2_1_2
Cell dimensions		
*a*, *b*, *c* (Å)	79.14, 79.14, 37.88	79.14, 79.14, 37.88
α, β, γ (°)	90, 90, 90	90, 90, 90
Resolution (Å)	35–1.85	35–1.77
Total reflections	74 533 (5123)	45 563 (2156)
Unique reflections	10 534 (653)	12 936 (702)
*R*_merge_	0.239 (0.345)	0.249 (0.337)
*I*/σ*I*	3.5 (2.9)	5.8 (2.2)
Multiplicity	7.1 (7.8)	3.5 (3.1)
Completeness (%)	98.5 (99.8)	95.3 (98.0)
Refinement		
*R*_work_/*R*_free_	0.2961/0.3081	0.2840/0.2910
Ramachandran (%) favoured allowed outliers	99 1 0	99 1 0
R.m.s. deviations		
Bond lengths (Å)	0.0077	0.0100
Bond angles (°)	1.4669	1.6641
*B* factor (Å^2^)	27.40	27.03
*MolProbity* clashscore	2.04	1.53
