# Supplementary material for: Design and fabrication of 3D-printed in situ crystallization plates for probing microcrystals in an external electric field
Source: J Appl Crystallogr. 2024 Apr 15;57(Pt 3):842–7. doi: 10.1107/S1600576724002140 (PMC11151662; doi:10.1107/S1600576724002140)
Supplement: Supplementary file 1 [file j-57-00842-sup1.pdf]

JOURNAL OF
APPLIED
CRYSTALLOGRAPHY

Volume 57 (2024)

Supporting information for article:

Design and fabrication of 3D-printed *in situ* crystallization plates for probing microcrystals in an external electric field

Krishna Prasad Khakurel, Michal Nemergut, Veronika Dzupponova, Kamil Kropielnicki, Martin Savko, Gabriel Žoldák and Jakob Andreasson

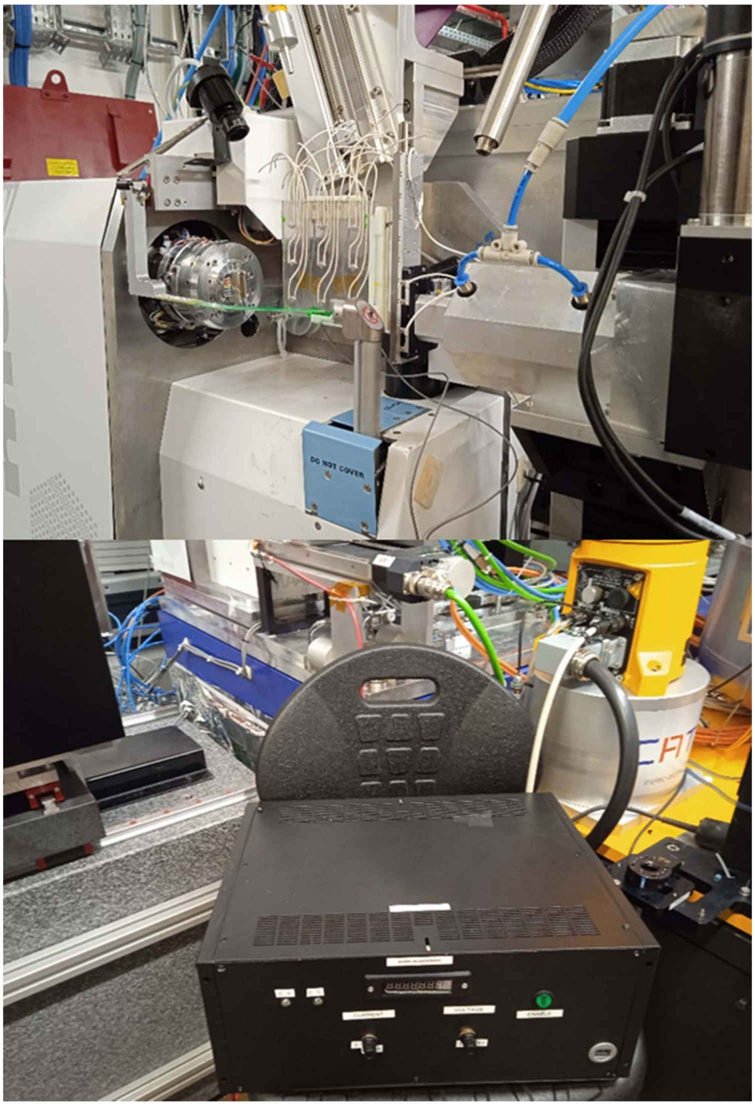

Supplementary Figure 1. A picture showing how the designed plates are connected to the power supply and mounted on the X-ray beamline.

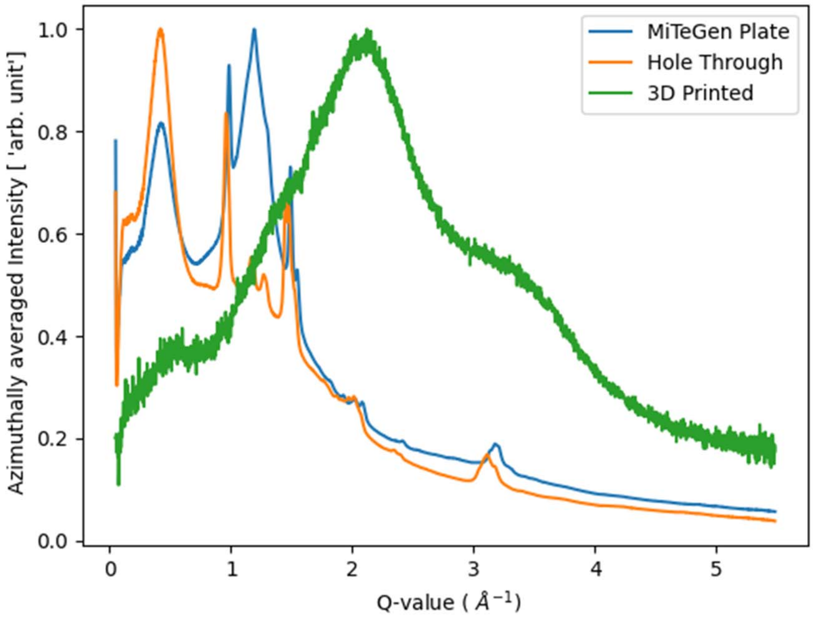

Supplementary Figure 2. A normalized azimuthal plot of the background collected from the MiTeGen plate, the hole through plate and the 3D-printed plate presented in the main article.
